# Exhibition to Celebrate 5 Years of the Bristol University Chair of Orthopoedics

**Published:** 1990-09

**Authors:** H. E. D. Griffiths


### AN EXHIBITION TO CELEBRATE THE FIRST FIVE YEARS OF THE CHAIR OF ORTHOPAEDIC SURGERY

At the completion of the first five years of the Zimmer Chair of Orthopaedic Surgery at the University of Bristol, it was decided to assemble an exhibition setting out some aspects of the research and development work that has been accomplished over the period. Professor Louis Solomon, the staff of the University Dept, and the orthopaedic Departments of the BRI, Frenchay and Southmead Hospitals mounted the exhibition in the Victoria Rooms, Clifton on Monday 25th June 1990 and in the words of the Vice-Chancellor Sir John Kingman, in the foreword to the programme, it gave many groups and individuals, who through their foresight and generosity brought the chair into being, the opportunity of seeing this work as a tribute to their efforts, and a record of continuing progress in the management of trauma and orthopaedic disease.

The exhibition was opened by Sir John Kingman and Professor Solomon spoke briefly on his view as to the direction orthopaedic research would take in the future. Mr John Pool, the chairman of the trustees of the Appeal for the Chair, spoke of the need for vigilance on the part of the trustees and members of the department to secure the financial future of the Chair in the face of budgeting stringency in the University generally.

Unfortunately space does not permit a discription of the individual exhibits but a list of their titles indicates the scope of and range of work already achieved during the first 5 years of the new chair of Orthopaedic surgery. The exhibition was unfortunately on view for only one evening and many who would have wished to see it were unable to do so.

The exhibition was set up under the following headings:

UNIVERSITY OF BRISTOL DEPARTMENT OF ORTHOPAEDIC SURGERY

BRISTOL ORTHOPAEDICS PAST AND FUTURE L Solomon, P Watkins

TEACHING AND TRAINING L Solomon

LOOKING INTO JOINTS R M Atkins

A MODEL ENVIRONMENT FOR MODELLING BONES A E Goodship, Professor Comparative Orthopadeic Research Unit, University of Bristol

WALKING DISABILITY AND ENERGY COSTS IN OSTEOARTHRITIS OF THE HIP A J Ward

MECHANISMS OF CARTILAGE DEGRADATION IN ARTHRITIS A P Hollander, R M Atkins, D Eastwood, P Dieppe, C Elson, Departments of Pathology, Orthopaedic Surgery and Rheumatology, University of Bristol

OSTEOARTHRITIS OF THE HIP: ONE DISEASE, SEVERAL DISORDERS L Solomon, B Bitounis, A Ward

LOOKING INTO BONES I Watt

CORTISONE PLUS ALCOHOL EQUALS BONE DEATH L Solomon

A RECIPE FOR ARTIFICIAL BONE V G Langkamer, L Solomon

WHAT IS A SLIPPED DISC AND WHAT CAUSES IT? M A Adams, P Dolan, D S McNally Comparative Orthopaedic Research Unit, University of Bristol

THE WAY FORWARD WITH BACKS P Dolan, M A Adams, Comparative Orthopaedic Research Unit, University of Bristol

A FRAME UP FOR FRACTURES R M Atkins

SOUTH WEST BONE TUMOUR SERVICE J H Dixon

CONGENITAL DISLOCATION OF THE HIP D M Eastwood, C F Wakeley

OSTEOPOROSIS L Solomon and the National Osteoporosis Society

TOTAL JOINT REPLACEMENT G Bannister and L Solomon

POST-OPERATIVE LOOSENING OF HIP REPLACEMENT PROSTHESES G Bannister, A Miles Department of Orthopaedic Surgery, University of Bristol and Department of Mechanical Engineering, University of Bath

'DO-IT-YOURSELF' BLOOD TRANSFUSION R S Majkowski, I C Currie, J H Newman

WINFORD ON ITS KNEES J H Newman

PRESSING TIMES FOR ARTHRITIC KNEES R Majkowski, R M Atkins

AIMING TO NAIL A FRACTURE R Majkowski, A Baker, R M Atkins, M Daniel

WOUND INFECTION AFTER ORTHOPAEDIC OPERATIONS G J S Taylor, G C Bannister, S Calder

H. E. D. GRIFFITHS

